# One-Dimensional Four-Layered Photonic Heterostructures: Analysis of Transmittance

**DOI:** 10.3390/ma18071433

**Published:** 2025-03-24

**Authors:** Amita Biswal, Harekrushna Behera, Dah-Jing Jwo, Tai-Wen Hsu

**Affiliations:** 1 Department of Communications, Navigation and Control Engineering, National Taiwan Ocean University, Keelung 202301, Taiwan; amitabiswal1988@gmail.com (A.B.); djjwo@mail.ntou.edu.tw (D.-J.J.); 2 Center of Excellence for Ocean Engineering, National Taiwan Ocean University, Keelung 202301, Taiwan

**Keywords:** photonic heterostructure, transfer matrix method, light propagation, bandgaps

## Abstract

The transmittance characteristics and the band structure of photonic heterostructures consisting of four distinct dielectric materials are analyzed using the transfer matrix method. An enhanced band structure of such crystals is discovered. It is shown that the band structure is strongly influenced by the arrangement of unit cells in the periodic building blocks of the crystals. The transmission spectra are evaluated for varying layer thicknesses and incident angles to investigate their impact on wave propagation. The symmetrical results for periodicities, sub-layer thickness, and oblique incident angles indicate robust bandgaps with blue shifting and enhanced transmission. Moreover, the periodicity in different cases, followed by the period, has also shown to have a great impact on the emergence of multiple bandgaps. The photonic bandgap and frequency are associated with the lattice elements of the unit cell, shifting naturally as a fundamental property of the structure, which has been achieved by the alteration of unit cells. Hence, the proposed photonic heterostructures offer significant potential for developing efficient band-stop and band-pass filters, facilitating their use in multi-functional integrated optical circuits within the Terahertz spectrum.

## 1. Introduction

One-dimensional (1D) photonic crystals (PCs) are artificial structures, composed of a periodic arrangement of two (or more) materials in the form of thin films with different refractive indices. A critical feature of these structures is the appearance of frequency intervals in the spectrum of impinging electromagnetic waves, with very low transmittance/high reflectance Yablonovitch [1] and John [2]. These intervals are called bandgaps. One-dimensional PCs with a sufficiently high refractive index contrast can act as omnidirectional reflectors, reflecting all polarizations of incident light from any angle across a selectable frequency range [3,4]. The photonic bandgap (PBG) is the scattering of multiple coherent light waves in the periodic structure [5,6,7]. A fundamental example of a PBG structure is the fiber Bragg grating (FBG), which has been widely implemented in modern lightwave communication systems. One-dimensional periodic photonic crystals serve as the foundation for various optical applications, including waveguides [8], reflectors [9], photovoltaic devices [10], sensors [11], optical filters [12], and emission control in micro-cavities [13]. Recent research has focused on the optical properties of 1D photonic crystals, highlighting their potential for studying material characteristics with photonic transmission [14,15,16,17].

In the concurrent investigations, the effective properties of the mixed crystals for the propagation of electromagnetic waves (EMWs) in the infrared range have been explored precisely [14,18,19]. One way to develop 1D PCs with enhanced and better controllable transmittance properties, suggested recently in refs. [20,21,22], is to combine two or three conventional binary photonic crystals, which form photonic crystal heterostructures (PCHs). The bonding of AlAs with the GaA substrate increased the breadth of the reflective peak and created a broader bandgap [23]. Further, GaAs/AlAs have been investigated in various optical and electrical states using the Wannier–Bloch integrating method and a randomly selected pattern of substrate width [24]. The bandgap of AlAs/GaP superlattices is studied using the full-potential linearized augmented plane-wave method [25]. The isothermal liquid-phase epitaxial technique has been used to examine the reflective characteristics of $Ga_{1-x}Al_{x}As$ piles in the infrared wavelength ranges [26]. Additionally, the band patterns of GaAs/AlAs are explored through the analysis of both transverse optical (TO) and longitudinal optical (LO) mode frequencies [27,28]. Another way to enrich transmittance spectra is to use 1D multi-periodic PCs with a super-cell consisting of four materials, arranged into periodic sub-cells (see refs. [16,29]). Furthermore, these structures show the properties of the PBG change when an extra, uniform layer is added to a typical, two-component PC, both in terms of location and thickness.

Additionally, inserting secondary layers to 1D PCs in certain circumstances may cause the PBGs in the gap map to vanish or arise. This is accomplished by breaking the periodicity of a typical PC known as a bi-periodic PC. The transmission properties of 1D binary composite PCs have been analyzed in the gigahertz (GHz) range [30]. Dadoenkova et al. [31] explored the PBG at gigahertz (GHz) frequencies and the magnonic bandgap at petahertz (PHz) frequencies in a 1D composite photonic system with a bi-periodic structure. This design is relevant for optoelectronic devices, particularly in antireflection coatings. Due to their multi-functionality, the unique characteristics of bi-periodic PCs can be exploited to develop precisely engineered superphotonic systems [32]. Furthermore, these photonic structures can be used to construct multipurpose combined optical circuits for several applications in the optoelectronic area of nanoscale materials [33,34]. Due to their considerable scientific relevance and dependability in numerous new fields of engineering, nanophotonic devices such as omnidirectional reflectors, narrowband optical filters, photovoltaic antireflective coatings, and ultrasensitive angle sensors [35] are highly recognized. For next-generation 6G applications, novel technologies are currently being developed with these heterostructure mechanisms in the terahertz (THz) range, encompassing modulation-doped field-effect transistors, heterojunction bipolar transistors, hot-electron transistors, resonant tunneling transistors, quantum-well lasers, and a range of photonic and quantum-effect devices [36,37]. According to the experts, the most exciting and dynamic areas of semiconductor physics and device technology are those involved in these architectures of multifaceted PCs with bi-periodic structures that have recently been devoted much attention.

Although researchers have paid attention to studying the 1D complex PC made of a bi-periodic photonic structure [29,38,39,40], there are a few studies in a four-component (quarternary) bi-periodic photonic system with symmetric and asymmetric arrangements of unit cells (or dielectric layers). In the current work, we investigate the transmittance of PCHs with building blocks made of binary and quaternary PCs, i.e., to some extent, combining the multi-periodic PCs and sub-cells to vary the spectrum of transmitted light. For studying the light spectrum of PCHs, four types of materials with dielectric properties are employed, which are placed periodically but with different orders of arrangements inside the PC. We have investigated the bandgap behavior of GaA/AlA-based PCs integrated with two additional ceramic oxides featuring different crystallographic structures, which are suspended in the air. The two ceramic oxides, Al_2_O_3_ and ZrO_2_, are chosen for photonic and electronic applications due to their high refractive index contrast, optical transparency, thermal stability, and mechanical strength. The suggested complex unit cell photonic system’s transmission behavior is theoretically explored using the transfer matrix method (TMM) [41]. The different physical parameters, like the periodicities, sub-layer thickness, and angle of incidence, are studied for the multi-periodic PC. Additionally, GaAs are susceptible to oxidation, affecting long-term stability, while AlAs are highly reactive to moisture, leading to degradation. Thermal expansion mismatches can induce strain, impacting structural integrity. These limitations pose challenges for practical deployment in some photonic applications. However, the quaternary design further enhances multi-bandgap formation, making these structures valuable for optical filters, waveguides, lasers, and photonic integrated circuits.

## 2. Transfer Matrix Method for Photonic Crystal Heterostructures

**Figure 1:**
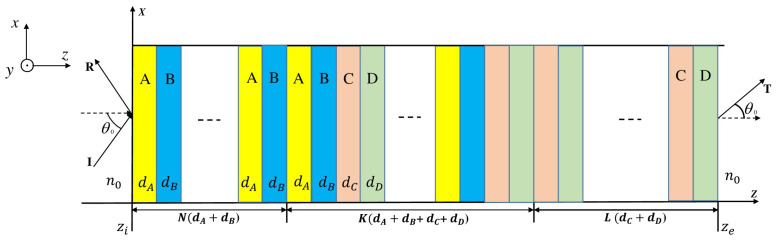
Schematic representations of a PCH, composed of a binary crystal ${\left(AB\right)}^{N}$, a quaternary crystal ${\left(ABCD\right)}^{K}$, and a binary crystal ${\left(CD\right)}^{L}$; the heterostructure is suspended in air.

A class of PCHs analyzed in this paper can schematically be represented as ${\left(AB\right)}^{N}{\left(ABCD\right)}^{K}{\left(CD\right)}^{L}$, where *A*, *B*, *C*, and *D* are four dielectric materials of refractive indices $n_{A}$, $n_{B}$, $n_{C}$, and $n_{D}$, with thicknesses $d_{A}$, $d_{B}$, $d_{C}$, and $d_{D}$, respectively. *N*, *K*, and *L* are the number of periods in each building block of the structure (see Figure 1). The structure is coupled to a homogeneous medium (air) at both incident and exit interfaces.

Here, we have considered the transverse electric (TE) mode of polarization to integrate the light wave into the structure. In the case of a TE wave, the electric field *E* is directed along the *y*-axis. For the dielectric layers, which are positioned within the x−y-plane, the *z*-axis is perpendicular to their interfaces. We now assume that an EMW of angular frequency ω, vacuum wave number k=ω/c, and c, the speed of light in vacuum, enter the heterostructure at normal incidence ($\theta_{0}=0$) from the left ($z<z_{i}$). Then, the coordinate parts of electric fields in the regions $z<z_{i}$ and $z>z_{e}$ can be expressed as

(1) $$E\left(z\right)=\exp\left[ik_{0}\left(z-z_{i}\right)\right]+r\exp\left[-ik_{0}\left(z-z_{i}\right)\right],\;\;E\left(z\right)=t\exp\left[ik_{0}\left(z-z_{e}\right)\right],$$

where $k_{0}=kn_{0}=\frac{\omega }{c}n_{0}$. The amplitude reflection *r* and transmission *t* coefficients are related by the *M* transfer matrix $M(z_{i},z_{e})$ (see ref. [42]), as follows:

(2) $$\left[\begin{array}{c} 1\;\\ r \end{array}\right]=M\left(z_{i},z_{e}\right)\left[\begin{array}{c} t\;\\ 0 \end{array}\right],\;M\left(z_{i},z_{e}\right)=L^{-1}\left(z_{i}\right)W\left(z_{i},z_{e}\right)\;L\left(z_{e}\right),$$

where

(3) $$L\left(z_{i}\right)=L\left(z_{e}\right)=\left[\begin{array}{cc} 1 & 1\;\\ ik_{0} & -ik_{0} \end{array}\right],\;W\left(z_{i},z_{e}\right)={\left(W_{A}W_{B}\right)}^{N}{\left(W_{A}W_{B}W_{C}W_{D}\right)}^{K}{\left(W_{C}W_{D}\right)}^{L}.$$

The W-matrix for each individual layer is

(4) $$W_{j}=\left[\begin{array}{cc} \cos(k_{j}d_{j}) & -\frac{1}{k_{j}}\sin\left(k_{j}d_{j}\right)\\ k_{j}\sin\left(k_{j}d_{j}\right) & \cos(k_{j}d_{j}) \end{array}\right],\;k_{j}=kn_{j}=\frac{\omega }{c}n_{j},\;j=A,B,C,D.$$

The transmittance and reflectance are then $T={\left|t\right|}^{2}$ and $R={\left|r\right|}^{2}=1-T$, respectively.

## 3. Numerical Results and Discussion

For the individual materials *A*, *B*, *C*, *D* of the heterostructure ${\left(AB\right)}^{N}{\left(ABCD\right)}^{K}{\left(CD\right)}^{L}$, we use the layers of GaAs, AlAs, Al_2_O_3_ and ZrO_2_ of thicknesses $d_{A}=62$ nm, $d_{B}=73$ nm, $d_{C}=150$ nm, and $d_{D}=70$ nm, respectively. The layer thickness of these materials in quaternary photonic crystals enables precise control over refractive index contrast and periodicity, allowing for the formation of multiple bandgaps at specific wavelength regions through constructive and destructive interference of light. The values of their refractive indices were obtained from refs. [43,44], where dispersion was also taken into account. These materials are transparent to light in the visible and near-infrared regions of the electromagnetic spectrum. They are compatible with thin-film deposition and other fabrication techniques used to construct one-dimensional photonic crystals (see, for example, [45,46]).

Their refractive indices can be found from their respective dielectric permittivities modeled as

(5) $$\begin{array}{cc} \; & \epsilon \left(\omega \right)=\epsilon_{\infty }\left\{1+\frac{\omega_{LO}^{2}-\omega_{TO}^{2}}{\omega_{TO}^{2}-\omega^{2}-i\omega \gamma }\right\}, \end{array}$$

where $\epsilon_{\infty }$, γ, $\omega_{LO}$ and $\omega_{TO}$ known as the static dielectric constant, photon damping constant, long-wavelength longitudinal-optical, and transverse-optical phonon frequencies. The values of these parameters are taken from refs. [14,16,28]. The refractive indices of Al_2_O_3_ and ZrO_2_ enhance PBG formation and optical confinement. Their transparency across UV to IR, oxidation resistance, and high-temperature stability make them ideal for optical coatings and extreme environments.

GaA/AlA quaternary photonic structures face challenges, like complex fabrication, high material costs, and sensitivity to oxidation and moisture, impacting stability. Thermal expansion mismatches can also induce strain, affecting structural integrity and performance. However, GaA/AlA-based quaternary photonic structures offer high refractive index contrast, enabling strong photonic bandgap formation and efficient light confinement. Their tunable bandgap, low optical loss in the near-infrared field, and strong nonlinear properties make them ideal for multi-wavelength filtering, optical switching, and frequency conversion. Additionally, they are compatible with semiconductor fabrication techniques, like molecular beam epitaxy (MBE) or metal–organic chemical vapor deposition (MOCVD), ensuring high-quality integration with optoelectronic devices.

### 3.1. Effect of Composite Dielectrics

**Figure 2:**
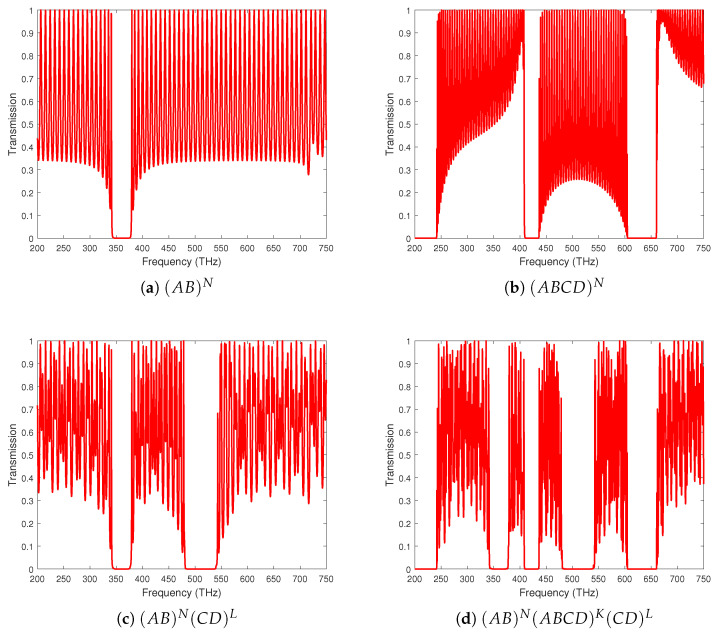
Light transmittance at normal incidence ($\theta_{0}=0^{\circ }$) through various photonic structures: (**a**) a quaternary crystal with an increasing number of periods *N*; (**b**) PCHs consisting of two binary crystals with an increasing number of periods *N* in the first one and second one; (**c**) PCHs consisting of two binary crystals at the edges with a fixed number of periods (N=L=40); and (**d**) a symmetric quaternary crystal in the middle with N=K=L=40.

The result in Figure 2a reveals the study of GaAs/AlAs without the addition of the multiple dielectric materials to the structure of PC [14]. It is worth noting that while changing the periodicity, more than one PBG has appeared in Figure 2b–d when considering the symmetric and asymmetric arrangements of complex unit cells. Additionally, the number of PBGs is higher for Figure 2d in comparison to Figure 2c, which verifies the sensitive nature of the semiconducting dielectric material arrangements inside the photonic lattice structure. The satellite transmission spectra of lower- and higher-frequency PBG exhibit symmetry concerning their central bandgap (360 THz), and the number of transmission stripes (or subpeaks) within each band is determined by the cell number. It is observed that the photonic structure with multiple dielectric materials has more than one bandgap compared to the absence of multilayers. The wider bandgaps with more numbers of unity transmission peaks indicate that the frequency is dependent on the PC’s unit cell composition, which can be used to design tunable stop-band filters.

### 3.2. Effect of Periodicity

**Figure 3:**
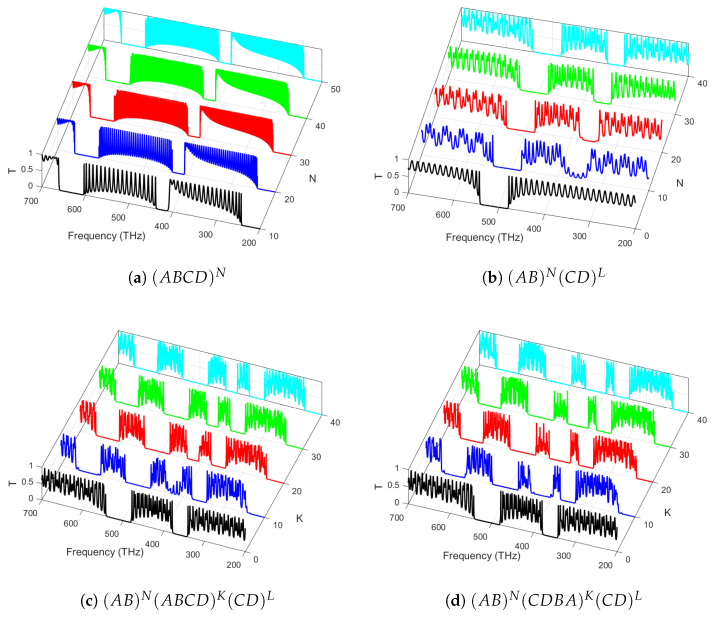
Light transmittance at normal incidence ($\theta_{0}=0^{\circ }$) through various photonic structures: (**a**) a quaternary crystal with an increasing number of periods *N*; (**b**) PCHs consisting of two binary crystals with an increasing number of periods *N* in the first one and a fixed number of periods (L=40) in the second one; (**c**) a symmetric quaternary crystal in the middle for an increasing number of period *K* at the edges with a fixed number of periods (N=L=40); and (**d**) an asymmetric quaternary crystal in the middle with an increasing number of *K* periods.

In Figure 3, the transmittance of light normally impinging on various photonic structures arranged from the above materials is investigated. In particular, in Figure 3a, we show the transmittance spectra for a conventional quaternary photonic crystal. As expected, the band edges become sharper with the increase in periods. In Figure 3b, we show the transmittance spectra of a PCH consisting of two binary crystals with a fixed number of periods. The bandgaps become wider and are shifted to higher frequencies. Further, in Figure 3c, we have considered a PCH consisting of the symmetric arrangement of a core quaternary crystal sliced between two binary crystals at the edges with a fixed total period count, creating a balanced photonic structure that enhances mode confinement and spectral tunability. However, for Figure 3d, an asymmetric quaternary crystal at the center, with an increasing number of periods, introduces gradient refractive index variations, leading to asymmetric light propagation, nonreciprocal effects, and tunable PBG properties. These configurations enable advanced control over light localization, wave manipulation, and the engineering of novel photonic states for next-generation optical devices. Moreover, the band structure becomes notably rich, appealing to the design of band-stop and band-pass optoelectronic filters.

### 3.3. Effect of Sub-Layer Thicknesses

**Figure 4:**
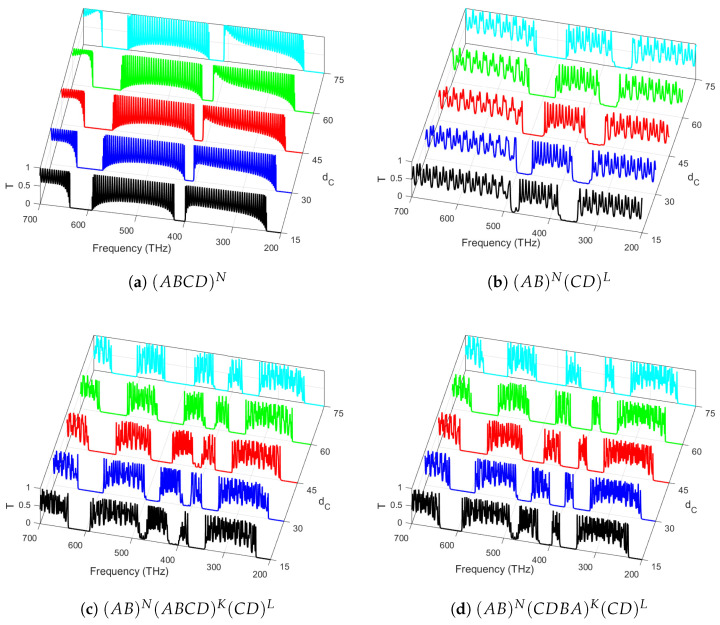
Light transmittance at normal incidence ($\theta_{0}=0^{\circ }$) through various photonic structures: (**a**) a quaternary crystal with an increasing number of periods *N*; (**b**) PCHs consisting of two binary crystals with an increasing number of periods *N* in the first one and a fixed number of periods (L=40) in the second one; (**c**) a symmetric quaternary crystal in the middle with a fixed number of periods (N=K=L=40); and (**d**) an asymmetric quaternary crystal in the middle with a fixed number of periods (N=K=L=40), respectively, for variable thicknesses of $d_{C}$.

In Figure 4a–d, the number of periods in all constituent parts of PCH is fixed. Moreover, we investigated the dependence of transmittance spectra on the thickness $d_{C}$ of the layer Al_2_O_3_. The blue shifting of bandgaps with a greater number of resonant peaks was seen when the layer thickness was increased for both symmetric (Figure 4c) and asymmetric (Figure 4d) arrangements of unit cells. It is worth mentioning that the $d_{C}$ (Figure 4b) has two bandgaps compared to Figure 4c,d. However, for Figure 4c,d, the number of bandgaps increases due to the increase in the thicknesses within a fixed frequency range for both symmetric and asymmetric arrangements of PCs. Further, as the layer thickness increases, the PBG remains at its maximum width. At the same time, its spectral boundaries shift toward longer wavelengths, governed by the effective refractive index modulation and the constructive interference conditions dictated by Bragg’s law. Bragg’s gap and frequency are influenced by the lattice composition of the unit cell, with frequency shifts arising as a fundamental consequence of the structural characteristics. Furthermore, the bandgap width changes depending on the specific relationships between the optical layer thicknesses ($n_{j}d_{j}$). The corresponding gaps in the frequency bands related to the wavelengths are due to the direct relation between λ and $n_{1}d_{C}$ [41]. The selection of optical layer thicknesses is based on the required transmission and reflection performance across different wavelength regions. Consistent layer thickness and material uniformity ensure stable bandgap properties, reducing scattering and losses. Precise control is important for optimal performance in optical filters, waveguides, and nonlinear devices. This feature highlights the tunable nature of filters, making them valuable for wavelength division multiplexer (WDM) applications and essential in optical communication.

### 3.4. Effect of Incident Angle ($\theta_{0}$)

**Figure 5:**
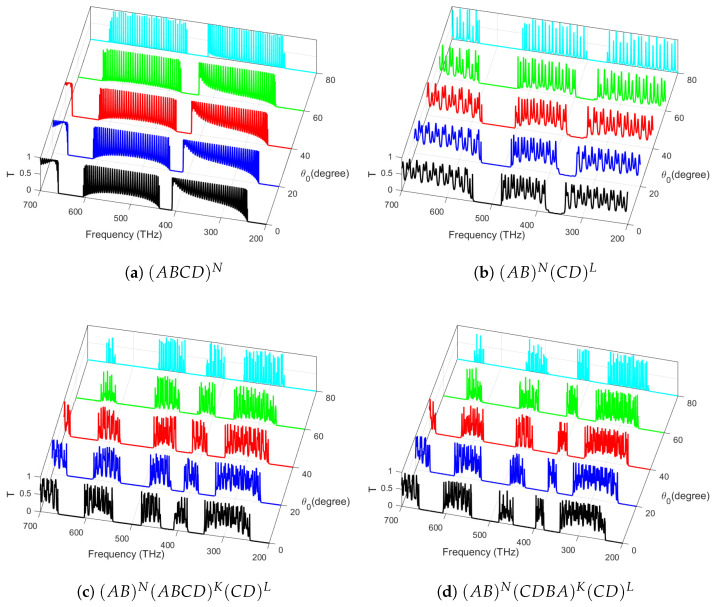
Transmission spectra of different photonic structures: (**a**) a quaternary crystal with an increasing number of periods *N*; (**b**) PCHs consisting of two binary crystals with an increasing number of periods *N* in the first one and a fixed number of periods (L=40) in the second one; (**c**) a symmetric quaternary crystal in the middle with a fixed number of periods (N=K=L=40); and (**d**) an asymmetric quaternary crystal in the middle with a fixed number of periods (N=K=L=40), respectively, with increasing values of $\theta_{0}=0^{\circ }$.

The transmission spectra *T* for different TE polarized oblique incidence angles in the multi-periodic photonic structure are explored in Figure 5a,b. The analysis reveals that increasing the incident angle enhances the transmission peak intensity and shifts the mode Bragg gaps toward higher frequencies. Furthermore, the dependence of the lattice constant on mode frequency introduces a blue shift in both gaps, offering a valuable advantage for the development of optical stop-band filters in advanced electronic applications. The PBG is at its largest, maintaining a nearly constant width while its borders gradually shift to higher wavelengths as the layer incident angle increases. However, for Figure 5c,d, it can be concluded that all Bragg gaps, in terms of breadth and position, have a modest reliance on the increasing values of the oblique incident angles concerned with the symmetric and asymmetric arrangements of PCH. The transmission inside the PBGs is shifted in the near-infrared region (NIR) as the angle of incidence increases. These effects collectively enhance the tunability and performance of photonic structures in advanced optical and electronic applications.

## 4. Conclusions

The transmittance spectra of 1D PCHs, composed of two semiconductor dielectrics and two ceramic oxides, are analyzed with the aid of the transfer matrix method. The transfer matrix approach is used in this investigation to analyze the photonic transmission spectra for a one-dimensional multi-periodic heterostructure PC composed of dielectric oxides. The transmission of the 1D PC for various parameters reveals the robust and blue-shifting bandgaps caused by the absorption of light waves in the fixed frequency range with alternating complex unit cells. The variation in periodicities also shows the robustness and the blue change of PBGs for different case studies following the period. Additionally, by altering the thickness of $d_{C}$ followed by changes in unit cells, the blue shifting of layer thicknesses is seen, demonstrating the relationship between the components of the structure and the mode’s frequency. As the oblique incident angle increases, the transmission peaks exhibit greater magnitudes, accompanied by a shift of mode Bragg gaps from lower to higher frequencies due to angle-dependent phase-matching conditions. Simultaneously, variations in the lattice constant influence the mode frequency, leading to a blue shift of both PBGs, which is an important phenomenon for controlling wave propagation in optical stop-band filter design. This blue shift arises from the interaction between the photonic crystal’s periodicity and the dispersion characteristics of the constituent materials. These properties enable their use in antireflection coatings and optical filters, which makes them essential in advanced photonic systems. As a result, the filter can now be dynamically tuned to adjust both the bandwidth and its spectral position. This presents a highly efficient approach for precisely controlling light propagation within embedded multi-periodic photonic structures. Symmetrical and asymmetric configurations offer new possibilities for designing advanced photonic systems with customized optical properties for waveguiding, filtering, and light manipulation.

We believe that the exploration of 1D multi-periodic heterostructured photonic crystals offers a promising avenue for advancing optical superstructure design. This study provides insights into the interaction between periodicity and wave propagation, which could contribute to the development of novel photonic devices. From a practical standpoint, the observed bandgap characteristics are instrumental in the engineering of optical stop-band filters, multi-functional integrated optical circuits, and omnidirectional reflectors, particularly in the terahertz frequency regime.

## Data Availability

The data supporting the findings of this study are provided within this article, as detailed in the figure captions and their corresponding discussions.

## Abbreviations

The following abbreviations are used in this manuscript:

1D	One-dimensional
PC	Photonic crystal
PBG	Photonic bandgap
FBG	Fiber Bragg grating
EMW	Electromagnetic waves
PCH	Photonic heterostructure
TO	Transverse optical
LO	Longitudinal optical
GHz	Gigahertz
PHz	Petahertz
THz	Terahertz
TMM	Transfer matrix method
TE	Transverse electric
WDM	Wavelength division multiplexer
NIR	Near-infrared region
